# Facile Preparation of Multifunctional Hydrogels with Sustained Resveratrol Release Ability for Bone Tissue Regeneration

**DOI:** 10.3390/gels10070429

**Published:** 2024-06-28

**Authors:** Wenhai Zhang, Li Zheng, Yi Yan, Wen Shi

**Affiliations:** 1 Orthopedic Department, Tianjin Hospital, Tianjin 300211, China; 2 Department of Biochemistry and Molecular Biology, University of Nebraska Medical Center, Omaha, NE 68198, USA; lizhengunmc@gmail.com; 3 Healthcare Security Office & Biomedical Engineering Lab, Union Hospital, Tongji Medical College, Huazhong University of Science and Technology, Wuhan 430023, China; 4 Mary & Dick Holland Regenerative Medicine Program, University of Nebraska Medical Center, Omaha, NE 68198, USA

**Keywords:** bone tissue engineering, resveratrol, anti-inflammation, anti-oxidative

## Abstract

Injectable hydrogels show great promise for bone tissue engineering applications due to their high biocompatibility and drug delivery capabilities. The bone defects in osteoporosis are usually characterized by an oxidative and inflammatory microenvironment that impairs the regeneration capability of bone tissues. To attenuate the reactive oxygen species (ROS) and promote bone regeneration, an anti-oxidative hydrogel with osteogenic capacity was developed in this study. The poorly water soluble, natural antioxidant, resveratrol, was encapsulated in thiolated Pluronic F-127 micelles with over 50-times-enhanced solubility. The injectable hydrogel was facilely formed because of the new thioester bond between the free thiol group in modified F-127 and the arylate group in hyaluronic acid (HA)–acrylate. The resveratrol-loaded hydrogel showed good viscoelastic properties and in vitro stability and was cyto-compatible with bone-marrow-derived mesenchymal stem cells (BMSCs). The hydrogel allowed for a sustained release of resveratrol for at least two weeks and effectively enhanced the osteogenic differentiation of BMSCs by the up-regulation of osteogenic markers, including ALP, OCN, RUNX-2, and COL1. Moreover, the hydrogel exhibited anti-oxidative and anti-inflammatory abilities through the scavenging of intracellular ROS in RAW264.7 cells and inhibiting the gene expression and secretion of pro-inflammatory cytokines TNF-α and IL-1β under LPS exposure. In summary, the results suggest that our multifunctional hydrogel loaded with resveratrol bearing osteogenic, anti-oxidative, and anti-inflammatory actions is easily prepared and represents a promising resveratrol delivery platform for the repair of osteoporotic bone defects.

## 1. Introduction

Osteoporosis is a very common disease, affecting hundreds of millions of people world-wide [1]. The degeneration of bone tissue in osteoporosis can lead to fractures and great pain in patients [2]. Although bone grafting can be used to treat osteoporotic defects, it is complicated by many incidences, such as surgical site infections, donor shortages, and immune rejections [3]. Pharmacotherapy suffers from either short-term lack of efficacy or serious long-term side effects [4]. There is a great need for better therapy in osteoporosis treatment. Osteoporosis is also considered a chronic inflammatory disease characterized by locally abundant ROS, proinflammatory cytokines, and M1 type macrophages, which substantially impede bone healing and repair [5]. Advances in tissue engineering offer new approaches to restore the balance of bone metabolism, aid bone tissue growth, and repair osteoporotic bone defects through targeted delivery of therapeutics to the defective site or the replacement of damaged bone regions [6,7]. Particularly, polymer-based injectable hydrogels, which are biocompatible materials with physical and chemical properties close to the extracellular matrix (ECM) of the human tissue microenvironment, have emerged as good platforms for cellular growth and differentiation and drug delivery to support tissue engineering and regeneration [8]. Hydrogels, with the ability to fit into defective regions with irregular shapes, have been widely studied as carriers of various therapeutics for the local treatment of bone diseases [9]. 

There is also growing interest and effort in the application of hydrogels for the treatment of osteoporosis, and huge progress has been made so far [10,11]. For example, Chen et al. recently developed a methacrylate γ-poly(glutamic acid)/methacrylated gelatin-based hydrogel loaded with both the strong anti-oxidant manganese dioxide (MnO_2_) and the fibroblast-activating protein inhibitor (FAPi) for the repair of the osteoporotic bone defects [12]. The hydrogel was found to reduce ROS and inflammation and enhance osteogenesis both in vitro and in vivo. Another bioactive hydrogel was made of crosslinked tetra-armed poly (ethylene glycol) (tetra-PEG) with the integration of short-chain chitosan (CS) and nanoparticulate hydroxyapatite (nHAp) [13], and was demonstrated to have osteogenic promoting potency and immunomodulation activity in vitro and an improved bone defect healing effect in an osteoporotic bone defect rat model. Li et al. developed nano-hydroxyapatite (n-HA)/resveratrol (Res)/chitosan (CS) composite microspheres to create an anti-inflammatory and osteogenic microenvironment by local sustained release of resveratrol (Res) [14]. Qu and Qian invented an injectable thermosensitive hydrogel poly (D, L-lactide)-poly (ethylene glycol)-poly (D, L-lactide) (PLEL) system containing Res and dexamethasone (DEX)-loaded carbonated hydroxyapatite to alleviate inflammatory responses and promote osteogenesis [15]. Although many different natural and synthetic polymers can be used to prepare hydrogels, hyaluronic acid (HA) is becoming a popular choice in bone tissue engineering because of its excellent biocompatibility and critical roles in bone formation [16]. Some recent studies regarding the application of HA-based hydrogels in tackling the osteoporosis problem suggest the great potential of HA hydrogels in the repair of small osteoporotic bone defects [10,17]. While most of them only showed enhanced osteogenesis effects, it would be ideal to develop an HA-based hydrogel with not only the osteo-inductive property but also anti-inflammatory and anti-oxidative abilities to achieve a more effective treatment of osteoporosis [5].

Res is a natural polyphenol molecule that is prevalent in grapes, berries, and red wines [18]. It has potent anti-oxidative and anti-inflammatory properties [19]. It can also promote osteogenic differentiation and angiogenesis, thus facilitating bone formation and repair [18]. Resveratrol treatment in mice was also found to alleviate osteoporosis [20]. However, resveratrol has low water solubility, leading to poor bioavailability after oral administration [21]. It is also susceptible to degradation caused by environmental stress conditions, such as heat, light, and oxygen [22]. One more challenge arises from the cytotoxicity of Res at high concentrations [23]. An efficient Res delivery system with a sustained release behavior is necessary to maintain the therapeutic effects of Res and protect it from degradation while avoiding the serious side effects. One appropriate way to address this issue is to load Res into micro/nanoparticles. Res has been prepared in the form of microspheres, solid lipid nanoparticles, micelles, and hydrogels, followed by consecutive evaluations for various tissue engineering applications, including wound healing, cartilage regeneration, and even osteoporotic bone regeneration [15,22,24,25,26]. We have successfully encapsulated Res into 3D-printed scaffolds to promote mandibular bone regeneration [27]. All these studies have confirmed that a functional Res carrier could not only achieve targeted and localized delivery of Res but also increase its biocompatibility and advance regenerative progression in tissue engineering.

However, a major hurdle for the clinical translation of those delivery systems is that they are not easy to prepare, thus resulting in great heterogeneity in the prepared carrier system [28]. It is envisioned that a facile preparation method resulting in better homogeneity should facilitate the implementation of Res delivery systems in patients. Our group has recently developed micelle-crosslinked hydrogel systems to deliver a variety of hydrophobic drugs, in which the hydrophobic drugs were first solubilized in micelles with good homogeneity followed by hydrogel formation between the micelles and other polymer precursors [29,30]. Those hydrogels are readily injectable and provide the controlled release of hydrophobic drugs from the hydrogels in situ. We tried to apply the same strategy for Res delivery and found that the FDA-approved amphiphilic polymer, Pluronic F-127, could serve as an ideal carrier for Res because it could encapsulate Res easily by forming micelles in water and substantially increase Res solubility [31]. Meanwhile, the F-127 can be derivatized with certain functional groups for subsequent hydrogel preparation [29]. Our approaches could simplify the design of an osteoconductive and anti-inflammatory hydrogel system with good control of the drug loading amount and efficiency, while in comparison, those previously mentioned anti-osteoporosis hydrogel-based approaches were much more complex and normally utilized the microspheres as drug carriers with limited drug loading content. 

Herein, an HA-based injectable hydrogel crosslinked by Res-loaded F-127 micelles with the ability to promote osteogenesis and reduce ROS and attenuate inflammation was developed. The rheological properties and stability of the hydrogel were first carefully characterized. The cytocompatibility and Res release behavior of the hydrogel were next evaluated. The effect of the hydrogel on osteogenic differentiation was determined on the bone-marrow-derived mesenchymal stem cell (BMSC). In the end, to delineate the anti-ROS and anti-inflammation potency, macrophage RAW264.7 cells were stimulated in vitro with lipopolysaccharide (LPS), and the intracellular ROS level, the gene expressions, and protein secretions of two pro-inflammatory cytokines, tumor necrosis factor (TNF)-α and interleukin (IL)-1β, were investigated with or without the hydrogel treatment. We envision that both osteo-inductive and anti-inflammatory effects can be simultaneously achieved by this facilely prepared hydrogel loaded with Res, in the hope of effectively repairing osteoporotic bone defects in the future. 

## 2. Results and Discussion

### 2.1. Fabrication of a Res-Loaded Injectable Hydrogel

**Figure 1:**
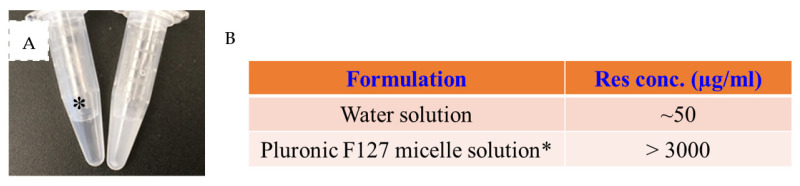
Pluronic F-127 enhanced the solubility of resveratrol. (**A**) Appearance of micelle loaded with resveratrol and free resveratrol in water (0.3 mg in 0.1 mL). (**B**) Estimated solubility of resveratrol in both formulations.* Eppendorf tube containing F-127 micelle loaded resveratrol.

To enhance the water solubility of Res, an FDA-approved and generally considered as safe ingredient, Pluronic F-127, was utilized. The resveratrol was encapsulated in F-127 micelles through the thin film hydration method [32]. As shown in Figure 1A, the 10% F-127 micelle solution with 0.3% (*w*/*v*) encapsulated resveratrol was clear and free of particles, while the same amount of free resveratrol in water was opaque and showed a significant number of precipitates. The encapsulation efficiency was over 95%. The DLS study showed the micelles had a Z-average size of 19 nm in PBS solution at room temperature, with a PdI of 0.20, suggesting good homogeneity. We roughly estimated the Res solubility in water and compared the solubilized resveratrol content under two conditions. It was found that the F-127 enhanced the solubility of Res by over 50 times (Figure 1B). Our result is consistent with previous studies of high loading efficiency and content achieved using F-127 to encapsulate Res [33,34]. A preliminary stability study of the Res-loaded F-127 micelles was also conducted by incubating the Res micelles at 37 °C for 2 weeks [34]. Neither the size nor the PdI of the micelles showed any significant change after 2 weeks, suggesting the F-127 as a promising carrier for the delivery of Res. 

Resveratrol micelles can still be washed away after in situ injection. So, we sought to prepare a hydrogel system that anchors the micelles in the hydrogel, which should allow for long-term retention of the Res micelles in the injection site [29]. The F-127 was first modified with free thiol groups using our reported method [29]. A nearly 70% grating ratio was achieved after the conjugation as shown in 1H-NMR, and the content of free thiol groups was about 0.1 µmole/g, based on the Ellman’s assay. 

The HA-acrylate conjugate was synthesized by reacting the acrylic anhydride with the HA backbone through an ester bond. The schematic of the synthetic strategy of HA-acrylate is shown in Figure 1A. Acrylic anhydride was used as the reactant because it minimized the reaction steps and resulted in fewer impurities after the reaction compared to the previously used method [35]. To our knowledge, this is the first instance of preparing HA-acrylate using acrylic anhydride, even though methacrylic anhydride was frequently used for the preparation of methacrylated HA before [36]. The 1H-NMR was performed to characterize the conjugates, and the grafting ratio was tunable by adjusting the initial feeding amount of acrylic anhydride. We found that an approximately 24% grafting ratio was achieved when the molar ratio between the acrylic anhydride and HA was 2:1 (Figure S1). After doubling the amount of acrylic anhydride in the conjugation reaction, the grafting ratio was increased to 44%, which is almost double. The 24% grafting ratio of HA-acrylate was used for further studies, as an abundant acrylate group may increase the cytotoxicity of the conjugated material. While a one-pot synthetic method for the preparation of acrylated HA has been reported [37], it still involves the handling of reactive and a moisture sensitive agent, acryloyl chloride, which is not feasible for many biomedical engineering labs. Our synthetic method was more straightforward and easier to follow with comparable conjugation efficiency [37]. Overall, we found using acrylic anhydride to be simple and effective in introducing acrylate moiety to the HA backbone. 

**Figure 2:**
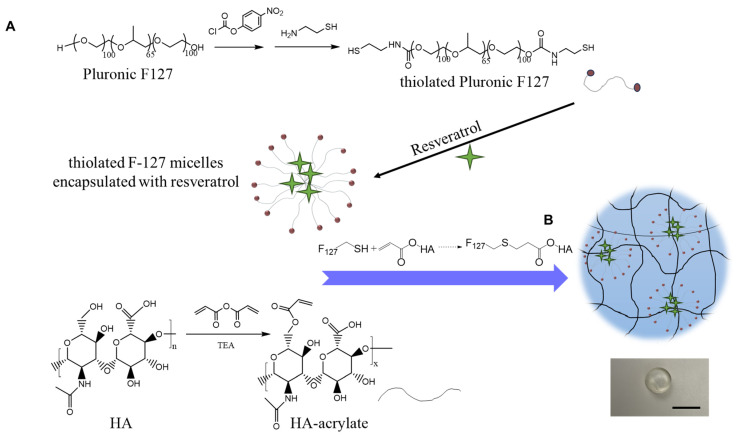
Schematic illustration of the preparation of F-127 micelle-crosslinked injectable HA hydrogel for sustained resveratrol delivery. (**A**) Synthetic strategy of two hydrogel precursors: thiolated F-127 and HA-acrylate; (**B**) schematic representation of the formation of the Res-loaded injectable hydrogel by simply mixing the two precursor solutions. Scale bar, 5 mm.

**Figure 3:**
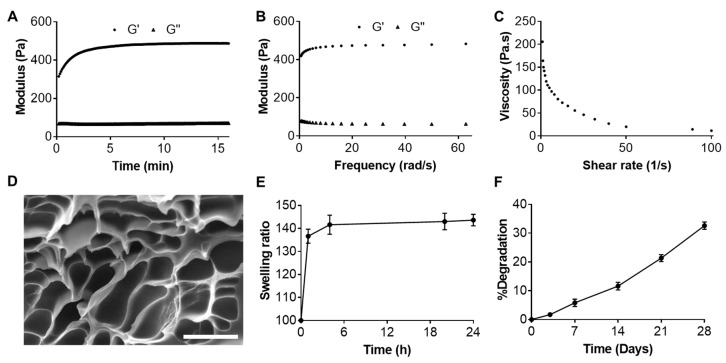
Physical characterization of resveratrol (Res)-loaded injectable hydrogel. (**A**) Time sweep study indicating the storage modulus (G′) and loss modulus (G″) change of the hydrogel after mixing the precursors. (**B**) Frequency sweep study indicating G′ constantly higher than G″ of the prepared hydrogel. (**C**) Viscosity of the injectable hydrogel with a shear rate increasing from 1 s^−1^ to 100 s^−1^. (**D**) SEM image of the Res-loaded injectable hydrogel. (**E**) Swelling of the Res-loaded hydrogel over 24 h. (**F**) Stability of the hydrogel within 28 days in PBS. Scale bar 20 µm.

The Res-loaded injectable hydrogel was easily prepared by mixing the thiolated F-127 solution (10% *w*/*v*) with the HA-acrylate solution (2% *w*/*v*) at a 1:1 volume ratio (Figure 2B). The gelation happened quickly (less than 1 min) at room temperature due to the thioester formation between the two precursors, but the hydrogel was very soft at the beginning. To further stabilize the hydrogel and enhance the crosslinking speed, we decided to incubate the hydrogel at 37 °C for 30 min. The incubation time was determined based on the time sweep assessment of the rheological behavior of the hydrogel (Figure 3A). 

### 2.2. Characterizations of Resveratrol-Loaded Hydrogel

The rheological properties of the Res-loaded hydrogel were next investigated. Although only 2% HA-acrylate was mixed with 10% thiolated F-127 in this study, our preliminary results indicated that the storage modulus (G′) of the Res hydrogel was dependent on the thiolated F-127 concentration (data not given). The storage modulus (G′) of the Res-loaded hydrogel was 300 ± 20 Pa after the mixing of the two precursors, and it gradually increased to 480 ± 30 Pa in 10 min (Figure 3A). The G′ remained constant after 10 min. The G′ was always higher than the loss modulus (G″, 80 ± 10 Pa) at moderate strains. The frequency sweep test implied a good elastic characteristic of the Res-loaded hydrogel as the G″ was consistently less than G′ at all the tested frequencies (Figure 3B). We also found the critical point when the G′ became less than G″ appeared at 75% strain.

The injectability of the hydrogel was represented by the substantial decrease in the viscosity after increasing the applied shear rate (Figure 3C). The viscosity was 205 Pa·s at a shear rate of 1 s^−1^ and gradually declined to 11.6 Pa·s at a rate of 100 s^−1^, suggesting a shear-thinning property of the hydrogel. This could come from the dynamic property of the F-127 micelles as the crosslinking agent. The Supplemental Video S1 also confirmed the good injectability of the hydrogel after being smoothly extruded through a 29-G needle with a syringe. 

**Figure 4:**
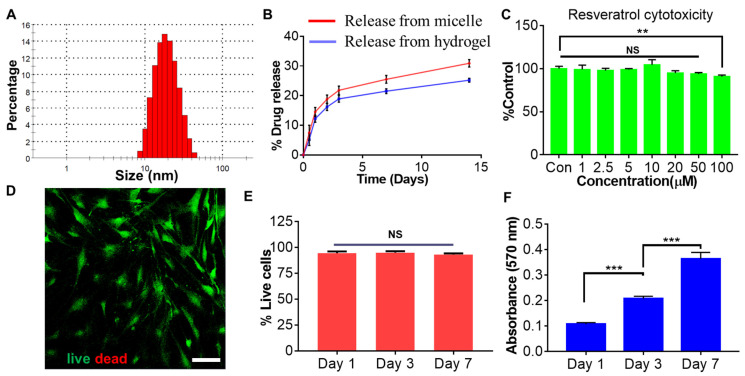
Characterization of Res-loaded micelles and the cytocompatibility of the resveratrol-loaded micelles-crosslinked hydrogel on BMSC. (**A**) Dynamic light scattering analysis of the Res-loaded micelles. (**B**) Resveratrol release profile from the micelle and the hydrogel over 14 days. (**C**) Cytotoxicity of resveratrol towards BMSC determined by an MTT assay after 48 h. (**D**) Representative live and dead staining of BMSC co-cultured the Res-loaded drug on day 7. (**E**) Viability of BMSC from day 1 to day 7. (**F**) Cell proliferation of BMSC in the presence of the Res hydrogel from day 1 to day 7. *n* = 3 in each replicate. ** *p* < 0.01, *** *p* < 0.001. NS, non-statistically different. Scale bar = 100 µm.

SEM imaging showed a highly porous structure of the Res-loaded hydrogel, with an average pore size of 15 µm. No Res particles or aggregates were found in the hydrogel in the SEM images, which agreed with the Res being solubilized in the form of micelles. At the same time, the hydrogel formation would not cause any precipitation of Res (Figure 3D). The hydrogel showed moderate swelling (~1.4 times) after immersion in PBS at 37 °C. This was probably due to the relatively high amount of the amphiphilic F-127 in the hydrogel, which turned hydrophobic at 37 °C and prevented the swelling of the polymer network (Figure 3E). The hydrogel was relatively stable in PBS, with about 35% degradation in 28 days (Figure 3F). In contrast, a previous study showed the hydrogel formed between 2% HA-acrylate and 2% thiolated HA was more prone to degradation and completely degraded within 28 days [37]. In the end, the drug release profile was determined using the dialysis method. Res was sustainedly released from the hydrogel, but the release rate was relatively slower than that from the micelle itself (Figure 4B). This was probably due to the additional barrier coming from the hydrogel network [30]. An average of 6.5% Res was released from the hydrogel within the first 12 h, with about 12% released in the first 24 h. However, only a total of 25.2% of the Res was released in a total period of 14 days. Although there was a burst release at the beginning, the cytotoxicity was still below the threshold. The slow and sustained release of Res during the whole stage was beneficial, as it could alleviate the toxicity concern from Res at a high concentration. 

### 2.3. Cytocompatibility and Osteogenic Activity of the Resveratrol-Loaded Hydrogel

The cytotoxicity of Res was first evaluated on human bone-marrow-derived mesenchymal stem cells (BMSCs). The cells showed over 95% viability at Res concentrations up to 50 µM but slightly decreased to 90% (*p* = 0.0082) at 100 µM Res (Figure 3C), implying a good safety profile of Res itself with BMSCs. Two hydrogel precursors, HA–acrylate and thiolated F-127, were both cyto-compatible with BMSCs, and the cells exhibited over 90% viability at even a 500 µg/mL concentration (Figure S2). Since HA and F-127 are both biocompatible materials, the results indicated minimal cytotoxicity with the functional group incorporation on the viability of BMSCs. 

Additionally, the influence of the Res-loaded hydrogel on the viability and proliferation of BMSCs was examined by a live/dead staining assay and an MTT assay at three time points. The BMSCs presented over 95% viability from days 1 to 7 when they were co-cultured with the Res-loaded hydrogel, as shown by the live/dead assay (Figure 4D,E). BMSCs continued to grow in the presence of the Res hydrogel for 7 days (Figure 4D). The MTT study revealed that the BMSCs almost doubled between days 1 and 3. By day 7, the BMSCs proliferated nearly 3.6 times as compared to day 1. The proliferation rate was comparable to that without the Res hydrogel co-culture. To sum it up, our results indicated good cytocompatibility of the Res-loaded hydrogel towards BMSCs. 

**Figure 5:**
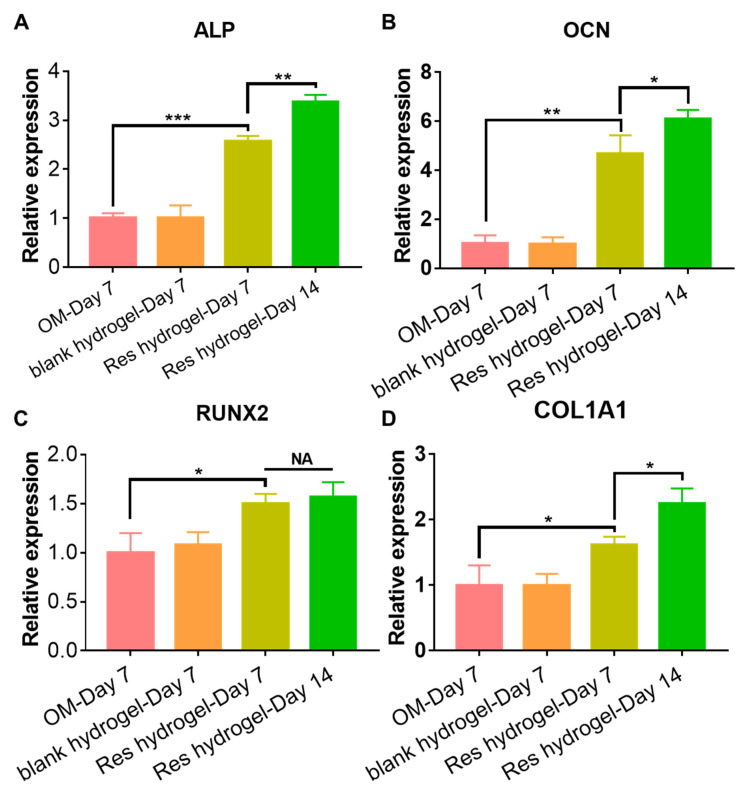
Res-loaded hydrogel enhanced the osteogenic differentiation of BMSC in osteogenic medium for 14 days. The expression of four osteogenic marker genes (**A**) ALP. (**B**) OCN. (**C**) RUNX2. (**D**) COL1A1 in osteogenic differentiation medium (OM), OM with a blank hydrogel, OM with the Res-loaded hydrogel. N = 3 in each replicate. * *p* < 0.05, ** *p* < 0.01, *** *p* < 0.001. NA, non-statistically different.

The osteogenic activities of the Res-loaded hydrogel were next investigated by evaluating the expressions of several early and late osteogenic marker genes, including alkaline phosphatase (ALP), osteopontin (OCN), runt-related transcription factor 2 (RUNX2), and collagen type 1 A1 (COL1A1) in BMSC cultured in the osteogenic medium with or without the Res-loaded hydrogel using RT-qPCR. All those genes play important roles in new bone formation. The significantly enhanced osteogenic activities in the Res-loaded hydrogel treatment group agreed well with many previous reports and our published work [24,27,38], indicating the released Res from the hydrogel was able to promote osteogenic differentiation of BMSC as early as 7 days (Figure 5). Particularly, the ALP gene expression was increased by nearly 2.5-fold (*p* < 0.001), and the OCN was increased by over 4.5-fold (*p* = 0.0016). In addition, the expressions were found to increase over time until day 14, except for RUNX2, although the RUNX2 expression at day 14 was still significantly higher than the osteogenic medium group. The underlying mechanism of Res’s osteogenic effect in human MSC was reportedly caused by its ability to upregulate the RUNX2 gene expression by directly activating the SIRT1/FOXO3A signaling [39]. In summary, we have clearly demonstrated the osteogenic potential of the Res-loaded hydrogel for bone tissue engineering.

### 2.4. Anti-ROS and Anti-Inflammation Properties of the Resveratrol-Loaded Hydrogel

**Figure 6:**
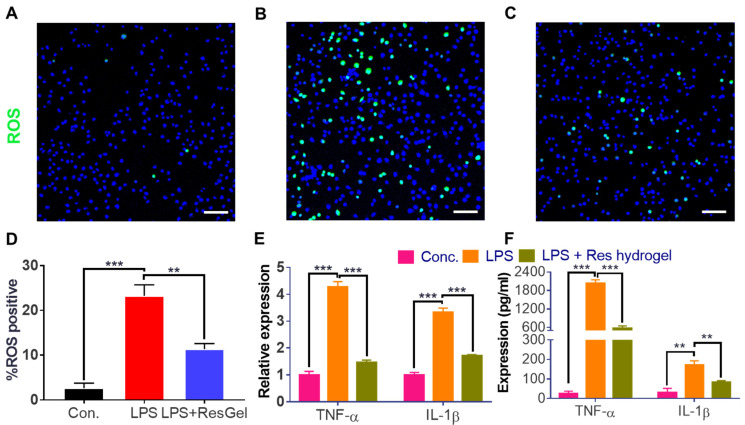
Characterization of anti-oxidation and anti-inflammation properties of the Res-loaded hydrogel. (**A**–**C**) ROS fluorescent imaging of RAW264.7 cells before LPS treatment (**A**) and after LPS treatment (**B**) as well as cells treated with the resveratrol-loaded hydrogel followed by LPS (**C**). (**D**) Quantitative results of the ROS-positive cell ratio in each group. (**E**) The mRNA expressions of pro-inflammatory cytokines TNF-α and IL-1β in different treatment groups. (**F**) The protein levels of pro-inflammatory cytokines TNF-α and IL-1β in different treatment groups, determined by ELISA. N = 3 in each replicate. ** *p* < 0.01, *** *p* < 0.001. Scale bar, 100 µm.

Res, itself, is a strong antioxidant and anti-inflammatory agent [15]. To evaluate whether the Res-loaded hydrogel could alleviate the oxidative stress on cells, macrophage cells RAW 264.7 were exposed to an LPS stimulus in the culture medium to induce oxidative and inflammatory responses [40]. The intracellular oxidative stress was detected by the ROS activatable probe, H_2_DCFDA. After H_2_DCFDA enters into the cytoplasm and gets activated by the elevated ROS intracellularly, it fluoresces green [41]. As shown in Figure 6A–D, more fluorescent cells (over 12-fold increase) were found in the LPS treatment group than the control cells. On the contrary, the Res hydrogel pretreatment group only showed a 4-fold increase compared to the control. A 65% reduction in the amount of ROS-positive cells was easily achieved, demonstrating that the Res hydrogel could effectively attenuate the oxidative stress on the macrophages in vitro. The potency mainly resulted from the Res ingredient, as the results showed little ROS scavenging effect from the blank hydrogel. The anti-oxidative property of Res agreed with many previous findings [42,43,44]. It was reported that the severity of osteoporosis is also relevant to the imbalance between the pro-inflammatory (M1) and anti-inflammatory macrophages (M2) [14]. Excess ROS in the osteoporotic bone defect region could hinder bone healing, and the deletion of excess ROS could favor the M2 macrophage polarization, thus promoting bone regeneration [45]. It would be interesting to see if this Res-loaded hydrogel could also influence the macrophage polarization and increase the M2/M1 ratio, in a future study. 

TNF-α and IL-1β are both pro-inflammatory cytokines that play critical roles in the regulation of bone homeostasis, and the elevated levels of TNF-α and IL-1β were presumably associated with decreased bone mass and greater fracture risk in patients with osteoporosis [46]. To delineate the anti-inflammatory value of the Res hydrogel, the effect of the LPS treatment and Res-loaded hydrogel pretreatment on the expression of the two genes in macrophages were first evaluated by RT-PCR. As shown in Figure 6E, TNF-α and IL-1β gene expression in RAW264.7 were upregulated by 4.3 and 3.3 times, respectively, after the LPS stimulus. In the group where the cells were pre-treated with the Res hydrogel, the gene expressions showed an average of 64% and 49% reduction (*p* < 0.001), correspondingly, compared to the LPS stimulus-only group. However, the gene expressions in the cells pre-treated with a blank hydrogel presented minimal changes, implying that the anti-inflammatory property of the Res hydrogel was from the Res component instead of the hydrogel itself. The secretion of the two pro-inflammatory cytokines was measured by ELISA. In correspondence to the gene expression changes, the secreted TNF-α and IL-1β were significantly increased after the LPS stimulus (Figure 6F). The amount of TNF-α elevated from an average of 24 pg/mL in the negative control to nearly 2000 pg/mL in the LPS exposure group. In the presence of the Res hydrogel, the amount of TNF-α only increased to an average of 570 pg/mL, which was almost a 70% reduction from the LPS-only group. In the meantime, the IL-1β level increased from an average of 30 pg/mL in the negative control to 170 pg/mL in the LPS group, while the level in the Res hydrogel pre-treatment group showed over a 50% decrease, with an average of 83 pg/mL. On the contrary, the TNF-α and IL-1β levels in the blank hydrogel group presented minimal differences compared to the LPS only group. Our study demonstrated that Res hydrogel could effectively suppress an LPS-induced inflammatory response, and its anti-inflammatory capability was comparable to some previously reported anti-inflammatory agents [42,47,48]. With these promising in vitro results, the efficacy of the Res-loaded hydrogel will be evaluated in a mouse osteoporotic model soon, in attempts of pain alleviation and the promotion of bone tissue regeneration.

## 3. Conclusions

In summary, a multifunctional Res-loaded hydrogel was facilely prepared by crosslinking the HA-acrylate with Res-loaded thiolated F-127 micelles. The hydrogel presented a storage modulus of around 500 Pa. It was relatively stable in vitro with only 35% degradation after a 28-day incubation period in PBS. The hydrogel was injectable through the 29-G needle. Res could be sustained released from the hydrogel with 25% of the initial amount released within 14 days, which resulted in good cytocompatibility of the hydrogel (over 95% viability co-cultured with BMSC was achieved). In vitro results demonstrated that the hydrogel also effectively scavenged intracellular ROS by 65%. It also enabled synergistic osteogenic and anti-inflammatory effects to potentially promote bone tissue regeneration. ALP expression was increased by 2.5-fold and OCN expression was improved by 4.5-fold. Pro-inflammatory gene expressions of TNF-α and IL-1β showed an average of 64% and 49% reduction, respectively. The versatility and feasibility of this hydrogel system suggests that it has great promise in the treatment of osteoporotic bone defects and facilitating the healing of defects. 

## 4. Materials and Methods

### 4.1. Synthesis of Acrylated Hyaluronic Acid (HA-Acrylate) Precursor 

A total amount of 50 mg of HA (290 kDa, Bloomage Biotech, Jinan, China) was completely dissolved in 5 mL of deionized (DI) water in a 50 mL flask, followed by the addition of 5 mL DMF (Acros Organics, Geel, Belgium). Acrylic anhydride (30 or 60 µL, TCI America, Portland, OR, USA) was quickly added to this mixture. The pH of the mixture was immediately adjusted to 8~9 with 1N sodium hydroxide (Fisher Chemical, Pittsburgh, PA, USA) solution. The flask was then cooled on ice, and the reaction mixture was stirred at 2~8 °C for 1 h. The mixture’s pH was occasionally adjusted to maintain its pH between 8 and 9. After that, the reaction flask was transferred to a cold room (4 °C), and the mixture was stirred for another 24 h. After that, the mixture was dialyzed against DI water in a cold room for 3 days to remove the impurities. The HA–acrylate conjugate was lyophilized and stored at −20 °C. A summary table regarding the information of all the used materials is listed below for reference.
**Agents (commercial)****Vendor & Purity****Amount used****Properties****Pluronic F-127**Sigma, >99%3.75 gNon-ionic copolymer, white powder, Mw ~ 12,700**Hyaluronic acid **Bloomage Biotech, >95%50 mgPolysaccharide, white powder, Mw ~ 290 kDa**Acrylic Anhydride**TCI, >95%30/60 µLVolatile liquid, Mw 126.1**p-Nitrophenyl chloroformate**Sigma, 96%0.48 gWhite powder, Mw 201.6**Cysteamine hydrochloride**Sigma, ≥98%11.3 mgColorless to very faintly yellow powder, Mw 113.6**Agents (synthesized)****Purity****Amount used****Properties****Thiolated Pluronic F-127**>90% pure, 70% grafting ratio 10% (*w*/*v*), 50 µLWhite lyophilized powder **Hyaluronic acid-acrylate **>90% pure, 24% grafting ratio2% (*w*/*v*), 50 µLWhite lyophilized powder 

### 4.2. Proton Nuclear Magnetic Resonance (^1^H NMR) Characterization

^1^H NMR spectra of the conjugates were characterized on a 500 MHz Bruker NMR system, and the results were analyzed using Topspin 4.0 software [49]. The conjugates were dissolved in deuterated water (D_2_O, Acros Organics, Geel, Belgium) at 6 mg/mL for NMR acquisition, with all chemical shifts referring to the D_2_O solvent peak (4.78 ppm) at 25 °C. The grafting ratio was determined using the ratio of the integral of alkene proton peak from conjugated acrylate moiety (5.8~6.5 ppm, -C*H*=C*H2*) to the integral of HA methyl proton peak (2.0 ppm, -C*H3*).

### 4.3. Preparation of Resveratrol-Loaded F-127 Micelles

Resveratrol (Res) is hydrophobic and has poor water solubility. A thiolated F-127 conjugate was previously synthesized in our lab and applied to encapsulate resveratrol to increase its solubility [29]. The micelles were easily prepared by the thin film hydration method [29,32]. In brief, the 10% (*w*/*v*) thiolated F-127 conjugate and 0.3% Res (TCI America) were first dissolved in methanol, separately. Then an equal volume of the Res solution was added to the thiolated F-127 solution in an Eppendorf tube. After vortex mixing the solution, the methanol was evaporated by blowing it with dry nitrogen, and a thin film was found on the surface of the tube. Next, the same volume of DI water as the initial F-127 solution was added to hydrate the film for 20 min at RT. The water solution was then sonicated for 20 min in a sonication bath, and the non-encapsulated Res was removed by centrifugation at 10,000× *g* for 5 min. The particle size and polydispersity (PdI) of the Res-loaded micelles was measured by a dynamic light scattering (DLS) analyzer (Malvern), following our previous method [30]. The non-encapsulated Res was re-dissolved in a 1:1 methanol/water mixture to determine the concentration based on the UV absorbance at 305 nm, with reference to the standard curve of the known concentration of Res prepared in 1:1 methanol/water. The encapsulation efficiency (EE) was calculated by the equation: EE = (Weight of feeding Res − Weight of non-encapsulated Res)/Weight of feeding Res × 100%. 

### 4.4. Fabrication of a Resveratrol-Loaded Injectable Hydrogel 

The Res-loaded injectable hydrogel was prepared by mixing 2 wt% of HA-acrylate, dissolved in a sodium chloride (NaCl) buffer (0.15 M, Fisher Chemical) at a pH of 7.4 ± 0.2 with Res-loaded thiolated F-127 (10%), dissolved in water, at a 1:1 volume ratio and stabilized at 37 °C for 30 min. The gelation occurred due to the thioether bond formation between the free thiol groups in the thiolated F-127 and acrylate groups in HA–acrylate. 

### 4.5. Rheological Characterization of the Resveratrol-Loaded Hydrogel 

The rheological properties of the Res-loaded hydrogel were characterized at 37 °C using a Discovery HR-2 rheometer (TA Instruments, New Castle, DE, USA) under several testing protocols [35]. At the beginning, 100 µL of the hydrogel precursor HA–acrylate (2% *w*/*v*) was transferred onto 20 mm parallel plates, and then 100 µL of Res-loaded thiolated F-127 solution (10% *w*/*v*) was quickly mixed with the HA–acrylate solution. It gelated quickly and the mixture formed a weakly cross-linked hydrogel before testing. The geometry gap was then set at 500 µm, and the strain was set at 10%. The storage modulus (G′) and loss modulus (G″) changes over 20 min were recorded in the time sweep study at the constant frequency of 2π rad/s to follow the gelation kinetics of the dynamic hydrogel. Next, the angular frequency was varied from 0.63 to 62.8 rad/s in the frequency sweep study to understand the viscoelastic properties. In the end, a flow sweep study was conducted on the hydrogel by linearly ramping up the shear rate from 1 to 100 s^−1^, and the viscosity was recorded under different shear rates. The strain amplitude sweep method (γ = 0.1–100%) with a constant frequency of 10 rad/s was conducted to track the storage and loss modulus change and the critical strain region for the stabilized hydrogel.

### 4.6. Swelling and Degradation Study

Every piece of freshly prepared hydrogel (100 µL) was immersed in 1 mL of PBS (Fisher Chemical, pH 7.4) in a 1.5 mL Eppendorf tube at 37 °C. A temperature of 37 °C was chosen because first it better mimics the in vivo condition with a body temperature of around 37 °C. Second, one of the composition F-127 is a thermos-responsive polymer with distinct behavior at 25 and 37 °C. The F-127 becomes hydrophobic at 37 °C. By incubating the hydrogel at 37 °C, it should have less swelling due to such increased hydrophobicity. At each time point, the hydrogel was removed from the PBS, and the excess water on the surface was wiped out. The hydrogel weights were measured (W_s_) on a balance before transferring them into 1 mL of fresh PBS. The initial weight was recorded as W_0_. The hydrogel swelling was monitored for 24 h and was determined with the equation: W_s_/W_0_ × 100%. The degradation ratio was determined using the equation: (W_0_ − W_s_)/W_0_ × 100%. Each time point had three replicate samples. 

### 4.7. In Vitro Drug Release Study

The release of Res from both the F-127 micelles and the injected hydrogels was evaluated by the dialysis method. Either every 50 µL of Res-loaded micelles or 100 µL of the Res-loaded hydrogel was put into a D-tube dialyzer (3.5 kDa, 250 µL volume) and dialyzed against 10 mL of PBS solution. At each time point, the releasate was collected, and the PBS solution was replaced with fresh solution. The released amount of Res at each time point was measured by the UV absorbance at 320 nm using the microplate reader (SpectraMax, Molecular Devices, San Jose, CA, USA). A standard curve of Res in PBS solution between 1 to 50 µg/mL was generated for reference.

### 4.8. Scanning Electron Microscopy (SEM) Imaging

The freshly prepared hydrogel was frozen in a −80 °C freezer followed by lyophilization in a freeze dryer, and the micro-structure of the freeze-dried hydrogel was studied by scanning electron microscopy (SEM, FEI Quanta 200, Hillsboro, OR, USA). The lyophilized powder was sprayed with a thin film of gold before its transfer to the SEM instrument for observation.

### 4.9. Cell Culture, Treatment, and Viability Studies

The human bone-marrow-derived mesenchymal stem cells (BMSCs) were purchased from the American Type Culture Collection (ATCC) and expanded in growth medium consisting of low-glucose Dulbecco’s Modified Eagle medium (DMEM, Gibco, Miami, FL, USA), 10% fetal bovine serum (FBS, Gibco), and 1% Penicillin-Streptomycin (P/S, Invitrogen, Carlsbad, CA, USA) supplemented with 1 ng/mL platelet-derived growth factor AA (PeproTech, Cranbury, NJ, USA) at 37 °C in a humidified incubator (Thermo Scientific, Waltham, MA, USA) containing 5% CO_2_ [27]. 

For the cytotoxicity study, 1 × 10^4^ /well BMSCs were seeded in each well of a 48-well plate, and the cells were attached to the plate overnight. On the next day, Res, at various concentrations between 1 to 100 µM, was prepared in cell growth medium. The cells in each well were incubated with the medium containing Res for 48 h. The control group was cultured in fresh growth medium. The cytotoxicity was analyzed by an MTT (3-(4,5-dimethylthiazol-2-yl)-2,5-diphenyltetrazolium bromide, Sigma, St. Louis, MO, USA) assay [50,51]. Each group contained 3 replicates.

To evaluate the hydrogel cytocompatibility study, BMSCs were harvested with TrypLE (Gibco), resuspended in growth medium, and seeded in each well of a 12-well plate (1 × 10^5^ cells). After overnight cell attachment, each 100 µL of freshly prepared Res-loaded hydrogel was added to the growth medium in 1 well of a 12-well plate. The cells continued to grow in the presence of the hydrogel for up to 7 days with the medium being changed every 2 days. At each pre-determined time point, live/dead staining was conducted using confocal microscopy (Zeiss 800, Zeiss, Oberkochen, Germany) to assess the viable cell ratio in three of the wells [52]. In the meantime, another three wells were analyzed by an MTT assay to determine the cell proliferation.

### 4.10. Mesenchymal Stem Cell Differentiation and RT-PCR Analysis

The BMSCs seeded in each well of a 12-well plate (1 × 10^5^ cells) were attached overnight and allowed to grow for another 3 days in the growth medium. Osteogenic differentiation was initiated at day 3 by replacing the growth medium with the osteogenic medium (OM) made of growth medium plus 100 nM of dexamethasone (Sigma), 10 mM of β-glycerophosphate (Sigma), and 50 μM of ascorbic acid (Sigma), and they were cultured for another 7 to 14 days [53]. In another two groups, 100 µL of either a freshly prepared blank hydrogel or a Res-loaded hydrogel was added into each well at the same time with the OM. The OM was replaced every second day while retaining the hydrogels.

At every harvest time-point, the total RNA was extracted from the cell monolayers using RNeasy mini-kits (QIAgen, Hilden, Germany), following the instruction manual. The total RNA was reverse transcribed into complementary DNA (cDNA) using an iScript cDNA synthesis kit (BioRad Laboratories, Hercules, CA, USA). Real-time PCR reactions were performed in a StepOnePlus™ Real-Time PCR System (Thermo Scientific, Waltham, MA, USA) using SYBR Green Supermix (Bio-Rad Laboratories, Hercules, CA, USA) with the housekeeping gene GAPDH as the internal control. The relative expression of each target gene was calculated using the comparative Ct (2^−ΔΔCt^) method. The primer sequence information for each target gene was provided in the Supplementary Materials.

### 4.11. Evaluation of Anti-Oxidative and Anti-Inflammatory Properties

The mononuclear macrophage leukemia cell line RAW264.7 was bought from ATCC and was cultured in DMEM/F-12 containing 10 % FBS and 1 % PS at 37 °C in an incubator containing 5 % CO_2_. To induce oxidative stress and inflammation in RAW264.7 cells in vitro, the cells were treated with lipopolysaccharide (LPS, 10 µg/mL, Sigma) for 8 h. For the determination of the anti-oxidative and anti-inflammatory ability of the hydrogel, the cells were pre-incubated with the freshly prepared Res-loaded hydrogel for 12 h before LPS exposure. 

A 2′,7′-dicholorodihydro fluorescein diacetate (H_2_DCFDA, Millipore, Burington, MA, USA) dye was used to analyze the intracellular oxidative stress (H_2_O_2_ levels) after LPS treatment [49]. The H_2_DCFDA probe was dissolved in DMEM/F-12 medium at a concentration of 5 µM to prepare the working solution. Cells in different treatment groups were incubated with the probe solution for 30 min and then in DAPI staining agent for 10 min. After removing the probe solution and washing them with PBS, the fluorescence of the cells was imaged with confocal microscopy (Zeiss 800). 

The supernatant from each treatment group was harvested, and the TNF-α and IL-1β contents were determined using an ELISA assay (RayBiotech, Peachtree Corners, GA, USA), following the manufacturer’s protocols. After that, the cells were washed with PBS, lysed immediately and extracted using RNeasy mini kits to collect the mRNA. The expression level of two pro-inflammatory genes, TNF-α and IL-1β, was evaluated by RT-qPCR, following the same procedure mentioned above. The primer information is listed in the Supplementary Materials.

### 4.12. Statistical Analysis

Quantitative results are collected from *n* = 3 replicates and represented as the mean ± standard deviation (SD). The GraphPad Prism 7 software was used for statistical analysis. The statistical significance was confirmed using the unpaired Student’s *t*-test and was indicated as below: * *p* < 0.05; ** *p* < 0.01; *** *p* < 0.001.

## Data Availability

The data presented in this study are available on request from the corresponding author.

## Supplementary Materials

The following supporting information can be downloaded at: https://www.mdpi.com/article/10.3390/gels10070429/s1, Figure S1: The grafting ratio of acrylate in HA can be determined by 1H-NMR in D_2_O; Figure S2: Cytocompatibility of synthesized hydrogel precursors: thiolated F-127(A) and HA-acrylate (B); Table S1: PCR primer sequences of target genes in relevant studies; Video S1: injection of dye colored hydrogel through a syringe needle set to the buffer solution.
