# Supplementary material for: Facile Preparation of Multifunctional Hydrogels with Sustained Resveratrol Release Ability for Bone Tissue Regeneration
Source: Gels. 2024 Jun 28;10(7):429. doi: 10.3390/gels10070429 (PMC11275495; doi:10.3390/gels10070429)
Supplement: Supplementary file 1 [file gels-10-00429-s001.zip › F-127 HA Resveratrol Hydrogel_supplementary.pdf]

Facile Preparation of Multifunctional Hydrogels with Sustained Resveratrol Release Ability for Bone Tissue Regeneration

Wenhai Zhang [1], Li Zheng [2], Yi Yan [3,*] and Wen Shi [4,*]

[1] Orthopedic Department, Tianjin Hospital, Tianjin 300211, China
[2] Department of Biochemistry and Molecular Biology, University of Nebraska Medical Center, Omaha, NE 68198, USA; lizhengunmc@gmail.com
[3] Healthcare Security Office & Biomedical Engineering Lab, Union Hospital, Tongji Medical College, Huazhong University of Science and Technology, Wuhan 430023, China
[4] Mary & Dick Holland Regenerative Medicine Program, University of Nebraska Medical Center, Omaha, NE 68198, USA

* Correspondence: yanyi@whuh.com (Y.Y.); donatellowenshi@gmail.com (W.S.)

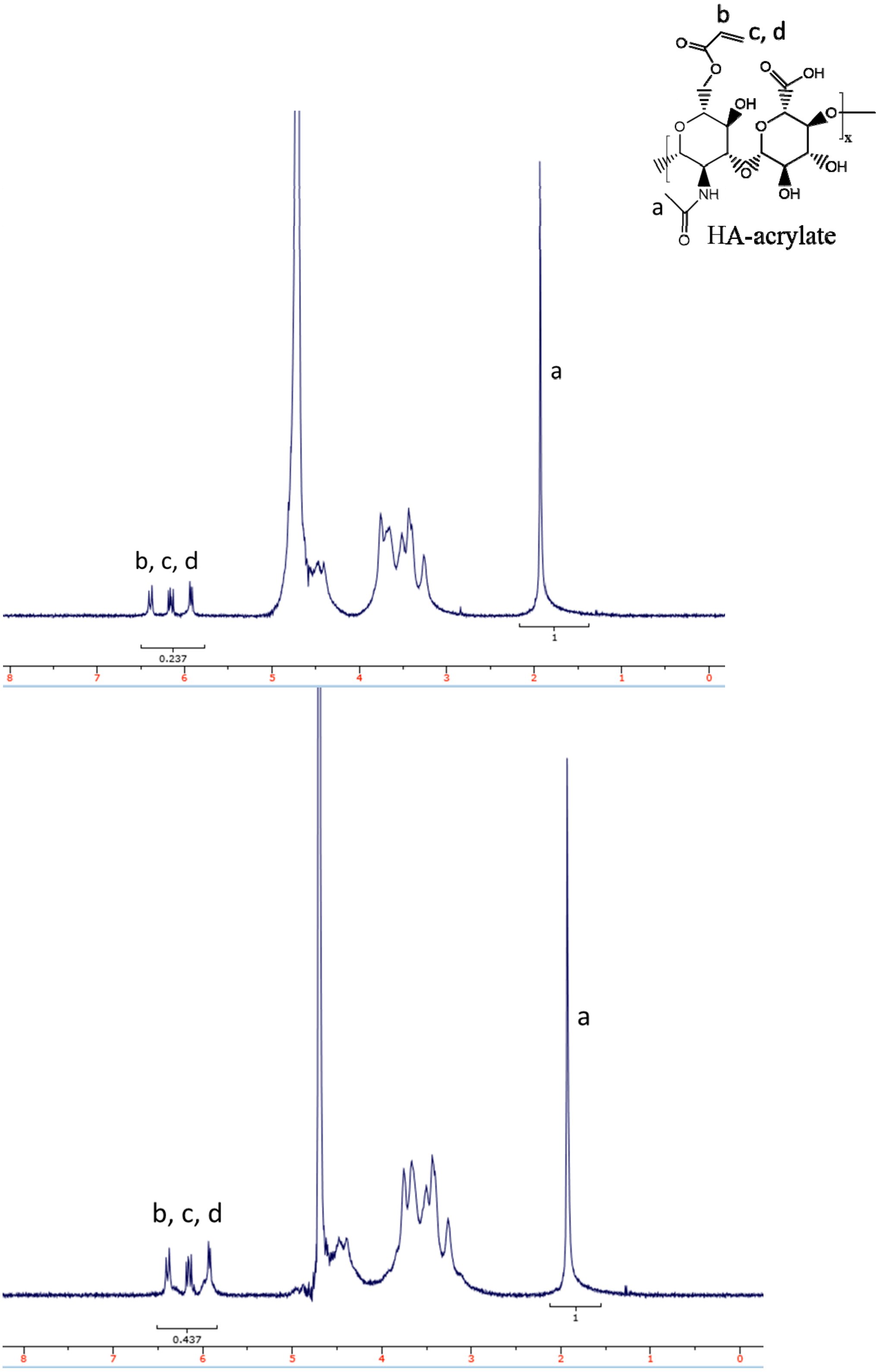

Figure S1: The grafting ratio of acrylate (top, low and bottom, high) in HA was determined by 1H-NMR in D2O.

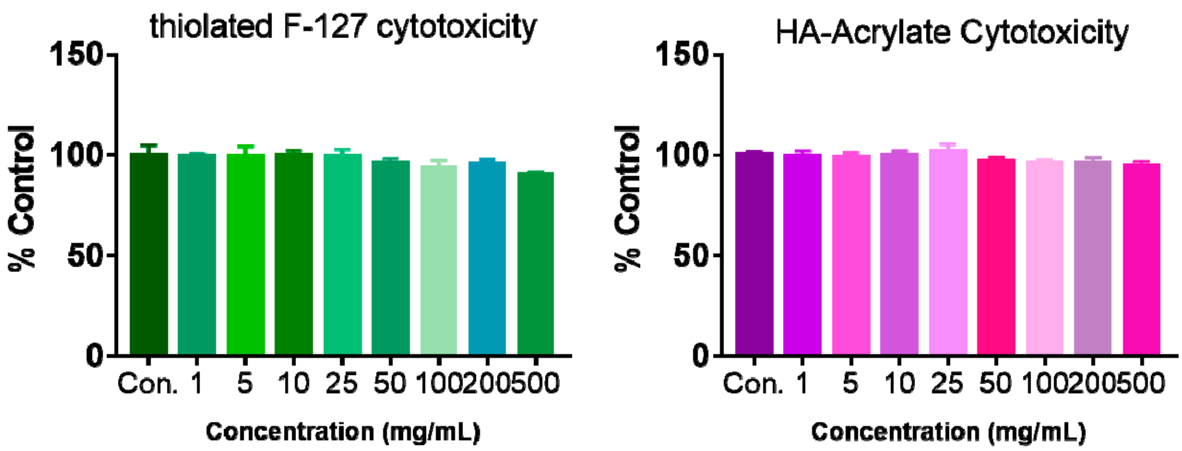

Figure S2. Cytotoxicity of thiolated F-127 and HA-acrylate.

Gene	Forward sequence	Reverse sequence
ALP	CAACGAGGTCATCTCCGTGATG	TACCAGTTGCGGTTCACCGTGT
OCN	GAAGGACAGTTATGAAACGAGT	AACATAGACATAACCCTGAAGC
RUNX2	ATGACGTCCCCGTCCATCCA	GGAAGGCCAGAGGCAGAAGTCA
COL1A1	TCCCCTCCACTCCTTCCCAAA	GGCCACTTGGGTGTTTGAGCA
GAPDH	TGAGCACCAGGTGGTCTCCTCTGA	TCCACCACCCTGTTGCTGTAGCCA

Gene	Forward sequence	Reverse sequence
TNF-α	GAGCTGTGGGGAGAACAAAAGGA	TTGGCCCTTGAAGAGGACCTG
IL-1β	GACCTTCCAGGATGAGGACA	AGCTCATATGGGTCCGACAG
GAPDH	AGAAGGCTGGGGCTCATTTG	AGGGGCCATCCACAGTCTTC

Table S1: Summary of PCR primer sequences of target genes.
